# Prevention of LPS-Induced Acute Lung Injury in Mice by Mesenchymal Stem Cells Overexpressing Angiopoietin 1

**DOI:** 10.1371/journal.pmed.0040269

**Published:** 2007-09-04

**Authors:** Shirley H. J Mei, Sarah D McCarter, Yupu Deng, Colleen H Parker, W. Conrad Liles, Duncan J Stewart

**Affiliations:** 1 The Terrence Donnelly Research Laboratories, Division of Cardiology, St. Michael's Hospital, University of Toronto, Toronto, Ontario, Canada; 2 Division of Infectious Diseases, McLaughlin-Rotman Centre for Global Health, Toronto General Research Institute, University of Toronto, Toronto, Ontario, Canada; 3 Institute of Medical Science, University of Toronto, Toronto, Ontario, Canada; 4 Department of Medicine, University of Toronto, Toronto, Ontario, Canada; 5 McLaughlin Centre for Molecular Medicine, University of Toronto, Toronto, Ontario, Canada; University College London, United Kingdom

## Abstract

**Background:**

The acute respiratory distress syndrome (ARDS), a clinical complication of severe acute lung injury (ALI) in humans, is a leading cause of morbidity and mortality in critically ill patients. ALI is characterized by disruption of the lung alveolar–capillary membrane barrier and resultant pulmonary edema associated with a proteinaceous alveolar exudate. Current specific treatment strategies for ALI/ARDS are lacking. We hypothesized that mesenchymal stem cells (MSCs), with or without transfection with the vasculoprotective gene *angiopoietin 1 (ANGPT1)* would have beneficial effects in experimental ALI in mice.

**Methods and Findings:**

Syngeneic MSCs with or without transfection with plasmid containing the human *ANGPT1* gene (pANGPT1) were delivered through the right jugular vein of mice 30 min after intratracheal instillation of lipopolysaccharide (LPS) to induce lung injury. Administration of MSCs significantly reduced LPS-induced pulmonary inflammation, as reflected by reductions in total cell and neutrophil counts in bronchoalveolar lavage (BAL) fluid (53%, 95% confidence interval [CI] 7%–101%; and 60%, CI 4%–116%, respectively) as well as reducing levels of proinflammatory cytokines in both BAL fluid and lung parenchymal homogenates. Furthermore, administration of MSCs transfected with pANGPT1 resulted in nearly complete reversal of LPS-induced increases in lung permeability as assessed by reductions in IgM and albumin levels in BAL (96%, CI 6%–185%; and 74%, CI 23%–126%, respectively). Fluorescently tagged MSCs were detected in the lung tissues by confocal microscopy and flow cytometry in both naïve and LPS-injured animals up to 3 d.

**Conclusions:**

Treatment with MSCs alone significantly reduced LPS-induced acute pulmonary inflammation in mice, while administration of pANGPT1-transfected MSCs resulted in a further improvement in both alveolar inflammation and permeability. These results suggest a potential role for cell-based *ANGPT1* gene therapy to treat clinical ALI/ARDS.

## Abstract 2

Using a mouse model of acute respiratory distress syndrome, Duncan Stewart and colleagues report that rescue with mesenchymal stem cells expressing human angiopoietin 1 can avert lung injury from lipopolysaccharide.

## Editors' Summary

**Background.:**

Critically ill people who have had an injury to their lungs, for example through pneumonia, trauma, or an immune response to infection, may end up developing a serious complication in the lung termed acute respiratory distress syndrome (ARDS). In ARDS, inflammation develops in the lung, and fluid builds up in the alveoli (the air sacs resembling “bunches of grapes” at the ends of the network of tubes in the lung). This buildup of fluid prevents oxygen from being carried efficiently from air into the blood; the individual consequently experiences problems breathing and can develop further serious complications, which contribute significantly to the burden of illness among people in intensive care units. The death rate among individuals who do develop ARDS is very high, upward of 30%. Normally, individuals with ARDS are given extra oxygen, and may need a machine to help them breathe; treatments also focus on addressing the underlying causes in each particular patient. However, currently there are very few specific treatments that address ARDS itself.

**Why Was This Study Done?:**

The researchers here wanted to work toward new treatment options for individuals with ARDS. One possible approach involves cells known as mesenchymal stem cells (MSCs). These cells are typically found in the bone marrow and have a property shared by very few other cell types in the body; they are able to carry on dividing and renewing themselves, and can eventually develop into many other types of cell. The researchers already knew that MSCs could become incorporated into injured lungs in mice and develop there into the tissue layers lining the lung. Some interesting work had also been done on a protein called angiopoeitin 1 (ANGPT1), which seemed to play a role in protecting against inflammation in blood vessels. Therefore, there was a strong rationale for carrying out experiments in mice to see if MSCs engineered to produce the ANGPT1 protein might “rescue” lung injury in mice. These experiments would be an initial step toward developing possible new treatments for humans with ARDS.

**What Did the Researchers Do and Find?:**

The researchers used a mouse model to mimic the human ARDS condition. This involved injecting the windpipe of experimental mice with lipopolysaccharide (a substance normally found on the outer surface of bacteria that brings about an immune reaction in the lung). After 30 minutes, the mice were then injected with either salt solution (as a control), the MSCs, or MSCs producing the ANGPT1 protein. The researchers then looked at markers of lung inflammation, the appearance of the lungs under a microscope, and whether the injected MSCs had become incorporated into the lung tissue.

The lipopolysaccharide brought about a large increase in the number of inflammatory cells in the lung fluid, which was reduced in the mice given MSCs. Furthermore, in mice given the MSCs producing ANGPT1 protein, the number of inflammatory cells was reduced to a level similar to that of mice that had not been given lipopolysaccharide. When the researchers looked at the appearance under the microscope of lungs from mice that had been given lipopolysaccharide, they saw signs of inflammation and fluid coming out into the lung air spaces. These signs were reduced among both mice treated with MSCs and those treated with MSCs producing ANGPT1. The researchers also measured the “leakiness” of the lung tissues in lipopolysaccharide-treated mice; MSCs seemed to reduce the leakiness to some extent, and the lungs of mice treated with MSCs producing ANGPT1 were no more leaky than those of mice that had never been injected with lipopolysaccharide. Finally, the MSCs were seen to be incorporated into lung tissue by three days after injection, but after that were lost from the lung.

**What Do These Findings Mean?:**

Previous research done by the same group had shown that fibroblasts producing ANGPT1 could prevent lung injury in rats later given lipopolysaccharide. The experiments reported here go a step further than this, and suggest that MSCs producing ANGPT1 can “rescue” the condition of mouse lungs that had already been given lipopolysaccharide. In addition, treatment with MSCs alone also produced beneficial effects. This opens up a possible new treatment strategy for ARDS in humans. However, it should be emphasized that the animal model used here is not a precise parallel of ARDS in humans, and that more research remains to be done before human studies of this sort could be considered.

**Additional Information.:**

Please access these Web sites via the online version of this summary at http://dx.doi.org/10.1371/journal.pmed.0040269.

- Medline Plus entry on acute respiratory distress syndrome, providing basic information about what ARDS is, its effects, and how it is currently managed
- ARDS Network from the US National Heart, Lung, and Blood Institute of the National Institutes of Health; the site provides frequently asked questions about ARDS as well as a list of clinical trials conducted by the network
- Information about stem cells from the US National Institutes of Health, including information about the potential uses of stem cells
- Wikipedia page about mesenchymal stem cells (note: Wikipedia is an internet encyclopedia anyone can edit)

## Introduction

The acute respiratory distress syndrome (ARDS), a clinically important complication of severe acute lung injury (ALI) in humans, is a significant cause of morbidity and mortality in critically ill patients [1–4]. Infectious etiologies, such as sepsis and pneumonia, are leading causes of ALI/ARDS [1,2]. Histologically, ALI/ARDS in humans is characterized by a severe acute inflammatory response in the lungs and neutrophilic alveolitis [1]. Inflammatory stimuli from microbial pathogens, such as endotoxin (lipopolysaccharide [LPS]), are well recognized for their ability to induce pulmonary inflammation, and experimental administration of LPS, both systemically and intratracheally, has been used to induce pulmonary inflammation in animal models of ALI [5–9].

The physiological hallmark of ARDS is disruption of the alveolar–capillary membrane barrier (i.e., pulmonary vascular leak), leading to development of noncardiogenic pulmonary edema, in which a proteinaceous exudate floods the alveolar spaces, impairs gas exchange, and precipitates respiratory failure [1,10,11]. Both alveolar epithelial and endothelial cell (EC) injury and/or death have been implicated in the pathogenesis of ALI/ARDS [1]. However, despite decades of research, few therapeutic strategies for clinical ARDS have emerged, and current specific options for treatment are limited [12–16]. ARDS continues to be an important contributor to prolonged mechanical ventilation in the intensive care unit, and ARDS-associated mortality remains high at 30%–50% despite optimal supportive care [1,13,14,16].

Marrow-derived stem or progenitor cells are being evaluated for the treatment of a number of diseases that currently have limited or no treatment options [17–22]. Recent studies have demonstrated that bone marrow-derived mesenchymal stem cells (MSCs) [23,24] can engraft in the injured lung [25,26] and even differentiate into lung epithelial cells in vivo [25,27,28]. MSCs may also exhibit immunosuppressive properties and have been suggested to be “immune-privileged”, and thus are protected from rejection, potentially permitting their use in allotransplantation [29–34]. Therefore, MSCs may have beneficial effects in their own right in the therapy of ALI [35].

Human angiopoeitin 1 (ANGPT1), a ligand for the endothelial-restricted receptor TEK tyrosine kinase (TEK; previously called TIE2) [36,37], plays an essential role in blood vessel maturation and stabilization during embryonic development. In the postnatal state, ANGPT1 maintains the normal quiescent phenotype of vascular ECs, protecting against vascular inflammation [38,39], reducing permeability [40–42], and promoting EC survival [43–46]. Given its anti-inflammatory, antipermeability, and endothelial-protective characteristics, we hypothesized that *ANGPT1* gene transfer may be beneficial in the treatment of ALI.

The combination of cell and gene therapies has proven successful in the treatment of experimental pulmonary hypertension [47–51]. This dual strategy not only allows direct targeting of the lung for clinical intervention, but also provides a site-specific source to release therapeutic proteins and/or other cellular products of interest by the retained cells. Therefore, the aim of this study was to evaluate the effect of MSCs alone or in combination with the vasculoprotective factor ANGPT1 on lung inflammation and injury induced by LPS in a murine model of ALI.

## Materials and Methods

### Characterization, Culture, and Transfection of MSCs

A frozen vial of murine MSCs (isolated from male C57Bl/6J mice; courtesy of Tulane Center for Gene Therapy, New Orleans, Louisiana, United States) was thawed and expanded as previously described [52]. Differentiation of MSCs was evaluated using a Mesenchymal Stem Cell Functional Identification Kit (R&D Systems, http://www.rndsystems.com/). Chondrogenic and osteogenic differentiation assays were performed in six-well Primaria plates (BD Biosciences, http://www.bdbiosciences.com/). The adipogenic assay was performed in eight-well Lab-Tek II Chamber Slide System (NUNC, http://www.nuncbrand.com/). MSCs were labeled with anti-mouse antibodies Sca-1 (clone E13–161.7), CD31 (clone MEC13.3), CD34 (clone RAM34), VCAM-1 (clone 429[MVCAM.A]), Flk1 (clone Avas 12a1), cKit (clone 2B8), CD45R (clone RA3-6B2), and CD11b (clone M1/70) and corresponding isotype controls purchased from BD Biosciences, and analyzed by flow cytometer (Cytomics FC500, Beckman Coulter, http://www.beckmancoulter.com/). MSCs used in all in vivo experiments were between passages 8 and 11. The full-length coding sequence of *ANGPT1* (1115 bp) was cloned into the expression vector pFLAG-CMV-1 (Sigma, http://www.sigmaaldrich.com/) as previously described [51]. MSCs were transfected by nuclear-targeting electroporation (nucleofection, Amaxa, http://www.amaxa.com/), SuperFect (activated dendrimer, Qiagen, http://www.qiagen.com/) or Lipofectamine (cationic lipids, Invitrogen, http://www.invitrogen.com/). For the in vivo study, nuclear-targeting electroporation was used to transfect MSCs with *ANGPT1* plasmid or empty vector plasmid. Human ANGPT1 protein expression was verified by ELISA (R&D Systems).

### Murine Model of LPS-Induced ALI

All animal procedures were approved in advance by the Animal Care Committee of St. Michael's Hospital (Toronto, Ontario, Canada). Carboxy-fluorescein diacetate, succinimidyl ester (Vybrant CFDA SE Cell Tracer Kit, Invitrogen) was used to label cells before injection into the animals. Aliquots of MSCs were analyzed by flow cytometry to confirm complete labeling. Female mice (19.9 ± 1.3 g) were anaesthetized and orally intubated with a sterile plastic catheter, and challenged with intratracheal instillation of 800 μg of LPS (E. coli 055:B5; Sigma) dissolved in 50 μl of normal saline. Saline, MSCs, MSCs transfected with pFLAG (MSCs-pFLAG), or MSCs transfected by plasmid containing human *ANGPT1* (MSCs-pANGPT1) (2.5 × 10^5^ cells, 100 μl total volume) were slowly infused via a jugular venous canula 30 min following LPS challenge. Human ANGPT1 protein expression was confirmed by ELISA for each batch of MSCs-pANGPT1 employed. Naïve mice (without LPS instillation) were injected with saline or MSCs to serve as controls for any inflammatory response that might result from the injected MSCs. After infusion, the canula was withdrawn, the vein ligated, and the incision sutured using silk suture. Mice were humanely killed at 15 min or 3 d after MSC treatment to collect tissues for analysis.

Due to the small size of the animals, two measurement groups were required to collect all tissue samples required. In one set of animals (*n* = 5 per group), lungs were lavaged three times with 1 ml of saline. Total cell counts were determined using a haemocytometer. Differential cell counts were determined on BAL smear slides that were stained with Hemacolor (EMD Chemicals, http://www.emdbiosciences.com/). Number of neutrophils was calculated as the percentage of neutrophils multiplied by the total number of cells in the BAL fluid sample. BAL fluid collected was then centrifuged at 800 *g*, and supernatant was collected for analysis of total protein, albumin, IgM, and cytokine/chemokine levels. All analyses were performed in a blinded fashion. The abdominal aorta and vena cava were severed at the diaphragm, and the right ventricle of the heart perfused with 10 ml of heparinized saline. The left lower lobe of the lung was snap-frozen, and later processed for lung homogenates. The rest of the lung was digested with dispase II (3.6 U/ml; Roche, http://www.roche.com/) into a single-cell suspension, according to a previously published protocol [53]. In another set of animals (*n* = 5 per group, except for the LPS/saline group [*n* = 9] and the LPS/MSCs group [*n* = 10]), blood was collected with a heparinized needle through cardiac puncture, and later centrifuged at 2,000 *g* to obtain plasma sample. Lungs were divided into two parts: the left lower lobe was collected as frozen tissue in OCT (Optimal Cutting Temperature; SAKURA FINETEK, http://www.sakura-americas.com/) and the rest was fixed by 4% paraformaldehyde for histology.

### Histopathology

Lung tissues (two cross sections of right lower lobe and one of right upper lobe) were fixed in 4% paraformaldehyde, embedded in paraffin, and cut into 5-μm thick sections. Sections were stained with hematoxylin and eosin, and images were taken with a Nikon Eclipse E800 microscope with a 40× objective. The average interalveolar septal thickness was quantified in a blinded fashion by measuring the thickness of all septae along a crosshair placed on each image (at least 150 septa per animal), using ImageJ software (National Institutes of Health; http://rsb.info.nih.gov/). For the lung injury score, images were evaluated by a investigator who was blinded to the identity of the slides (WCL) following a previously published scoring system [54].

### Measurement of Albumin, IgM, Cytokines, and Chemokines

Albumin and IgM levels in BAL fluid samples were measured using with a murine-specific albumin ELISA kit (ALPCO Diagnostics, http://www.alpco.com/) and a murine-specific IgM ELISA kit (Bethyl Laboratories, http://www.bethyl.com/), respectively. Cytokine levels (IFN-γ, TNF-α, IL6, and IL1β) in BAL fluid were measured with murine cytokine-specific Quantikine ELISA kits (R&D Systems). Chemokine levels in BAL fluid, and cytokine/chemokine levels in lung homogenates and plasma, were measured by multiplex immunoassay (Luminex 100, Luminex, http://www.luminexcorp.com/) using cytokine-specific bead kits (R&D Systems). Lung homogenates were processed according to a previously published protocol [55], and all results were normalized (25 μg of total protein per sample) with individual protein concentration measured from lung homogenate samples by Bradford assay (Bio-Rad, http://www.bio-rad.com/).

### MSCs Rentention Study

Mouse lung was digested into single cells using dispase II enzyme (3.6 U/ml, Roche) according to a previously published protocol [53]. The number of total cells recovered was determined by using a hemocytometer. Isolated cells were analyzed by a flow cytometer (FC500, Beckman Coulter) with a minimal collection of 30,000 events per sample. Cell nuclei were counterstained with nuclear dye TO-PRO-3 (Invitrogen), and confocal microscopic images were collected with a Leica TCS SL laser scanning confocal microscope.

### Statistics

Data in figures are represented as individual data points in a vertical dot plot, with a line to indicate the mean, except data for surface marker expression on MSCs, and retention of injected MSCs, in which data are represented as bar graphs showing mean ± standard error of the mean (SEM). Lung injury score and septal thickness data are shown in tabular form (mean ± SEM). Differences between the treated groups versus the injured group (LPS/saline) were assessed using a one-way ANOVA (with post hoc comparisons using Dunnett test) with statistic software (GraphPad Prism version 4.00; http://www.graphpad.com/). A value of *p* < 0.05 was considered statistically significant.

## Results

### Characterization and *ANGPT1* Transfection of MSCs

**Figure 1:**
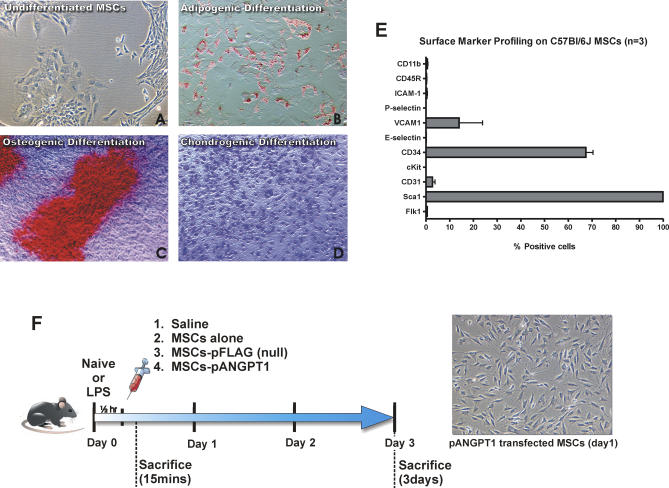
Characterization of MSCs Isolated from C57Bl/6J Mice, and Experimental Design for In Vivo Study (A) Undifferentiated MSCs (P8) were seeded in a T75 flask at a density of 100 cells/cm^2^ and found to expand readily in a fashion that started from a single cell to colony. (B) Staining with oil red-O was used to detect MSCs that differentiated into adipocytes, identified by perinuclear red staining of fat globules. (C) Staining with alizarian red was used to detect MSCs that differentiated into osteocytes. (D) MSCs that differentiated into chondrocytes were stained with toluidine blue. Photomicrographs were obtained with a 10× (A and C) or a 20× (B and D) objective using a Nikon Eclipse TS100 inverted microscope. (E) Flow cytometry was performed for surface marker expression on cultured MSCs. All data are presented as mean ± SEM. (F) C57Bl/6J mice initially received LPS by intratracheal instillation, followed by intravenous injection 30 min later with saline, cultured MSCs, MSCs-pFLAG, or MSCs-pANGPT1. Mice were then sacrificed 15 min and 3 d after to assess cell retention, or 3 d after to evaluate the therapeutic efficacy. A photomicrograph of MSCs-pANGPT1 was obtained using a Nikon Eclipse TS100 inverted microscope with a 10× objective 1 d after transfection and prior to injection into animals.

Murine MSCs were demonstrated to differentiate into three predominant mesenchymal lineages: adipocytes, osteocytes, and chondrocytes (Figure 1A–1D). The cells were uniformly positive for the stem cell surface marker Sca1 by flow cytometry (Figure 1E). Subpopulations of MSCs were CD34^+^ and VCAM1^+^, as previously reported for murine MSCs from the C57Bl/6J background [52]. The cells were negative for both CD11b (monocyte marker) and CD45R (leukocyte marker).

Flow cytometric analysis of MSCs transfected by nuclear-targeting electroporation resulted in ∼55% greater GFP expression in live cells compared to transfection with either SuperFect or Lipofectamine (unpublished data). At 24 h after nucleofection with pANGPT1, ANGPT1 protein (724 ± 283 pg/ml) was detected in the culture supernatant (from 5 × 10^5^ cells), and levels were sustained for more than 5 d (Figure S1; detailed methods described in Protocol S1). Phosphorylation of the TEK receptor, mediated by human ANGPT1 protein expressed using the same plasmid, has been previously validated by our group [51]. Nontransfected or null-transfected (empty vector) MSCs produced no detectable ANGPT1 protein.

### Effect of MSCs Alone or Transfected with *ANGPT1* on Acute LPS-Induced Pulmonary Inflammation

**Figure 2:**
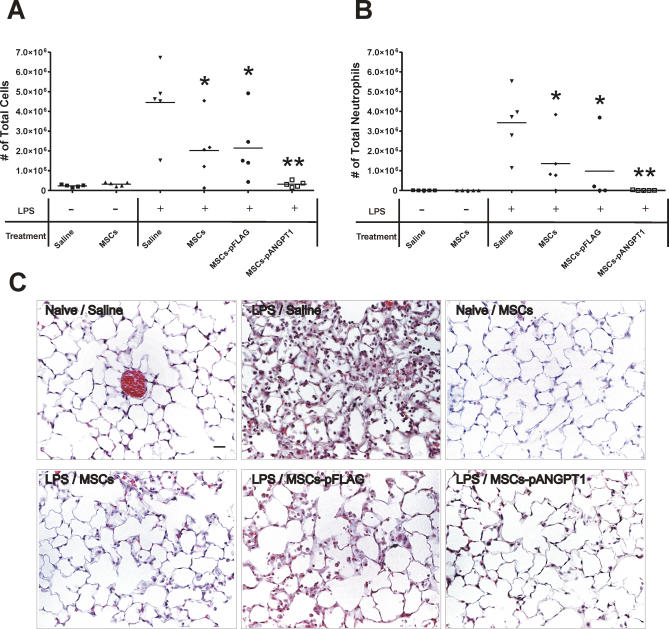
Therapeutic Potential of MSCs, Alone or Transfected with pANGPT1*,* on LPS-Induced Lung Inflammation in Mice (A and B) Total cell (A) and neutrophil (B) counts were performed on BAL fluid to evaluate lung airspace inflammation. There was a 19-fold increase in total inflammatory cells in BAL fluid collected 3 d after LPS, which was reduced by 53% in MSCs-treated mice (non-/null-transfected), and by 96% with MSCs-pANGPT1. Group comparisons were analyzed by one-way ANOVA with Dunnett post hoc test. *p < 0.05 and **p < 0.01, compared between LPS/saline versus each treated group (MSCs, MSCs-pFLAG, or MSCs-pANGPT1). *n* = 5 per group. (C) Histological evaluation of therapeutic potential of MSCs and MSCs-pANGPT1 on LPS-induced lung injury in mice. Representative images of hematoxylin and eosin stained lung sections from six experimental groups. Lungs were fixed with 4% paraformaldehyde, embedded in paraffin, and then cut into 5-μm thick sections before being stained. Photomicrographs were obtained with a Nikon Eclipse E800 microscope with a 40× objective. Scale bar = 20 μm.

LPS was administered to mice followed by an injection of saline, MSCs alone, or MSCs transfected with pFLAG (null) or *ANGPT1* plasmid (MSCs-pANGPT1) (Figure 1F). The total inflammatory cell count in the BAL fluid was increased approximately 20-fold at day 3 following administration of LPS, mainly attributable to an increase in neutrophils (around 80% of total cells in LPS/saline group). Treatment of animals with MSCs alone significantly reduced the total cell and neutrophil counts in BAL fluid (Figure 2A and 2B, *p* < 0.05 compared to LPS/saline group). Treatment with MSCs-pANGPT1 further reduced the BAL fluid cell count to a level similar to control, naïve mice (*p* < 0.01 for total cells and neutrophils counts compared to LPS/saline group). Substitution of skin fibroblasts or unfractionated bone marrow cells for MSCs did not prevent the observed LPS-induced increase in BAL fluid cell counts (unpublished data).

Histological assessment of lung sections 3 d after the administration of LPS revealed evidence of marked inflammatory infiltrates, interalveolar septal thickening, and interstitial edema (Figure 2C). MSCs alone reduced airspace inflammation, which was more apparent in mice treated with MSCs-pANGPT1. Morphometric analysis measuring interalveolar septal thickness showed a modest increase in the LPS alone group, with a significant reduction in animals receiving MSCs (Table 1). Severity of lung injury was also scored using a semiquantitative histopathology score system [54], which evaluates lung injury in four categories: alveolar septae, alveolar hemorrhage, intra-alveolar fibrin, and intra-alveolar infiltrates. Although treatment with MSCs or MSCs-pANGPT1 tended to reduce lung injury scores (Table 1), the observed differences did not reach statistical significance.

**Table 1:**
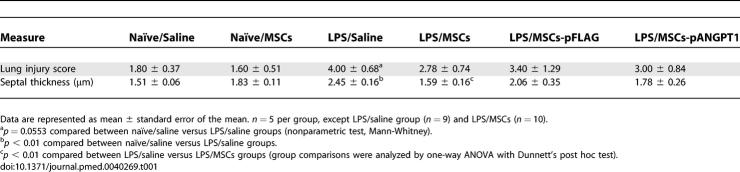
Lung Injury Score and Septal Thickness in Mice with LPS-Induced ALI

**Figure 3:**
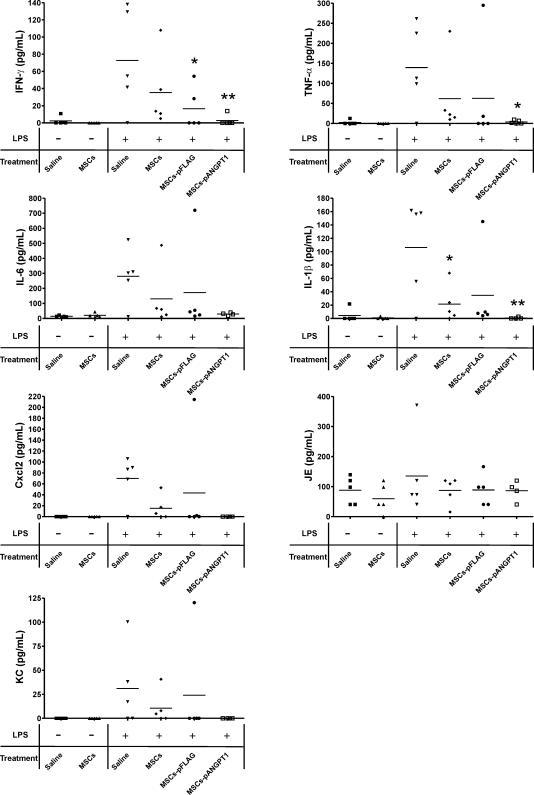
Levels of Proinflammatory Cytokines and Chemokines in BAL Fluid Levels of the proinflammatory cytokines IFN-γ, TNF-α, IL6, and IL1β in BAL fluid were measured using ELISA. In addition, chemokine levels (Cxcl2, JE [murine homolog of human CCL2], and KC [murine IL8 homolog]) in BAL fluid were measured by multiplex immunoassay. Group comparisons were analyzed by one-way ANOVA with Dunnett post hoc test. *p < 0.05 and **p < 0.01, LPS/saline versus each treated group (MSCs, MSCs-pFLAG, or MSCs-pANGPT1). *n* = 5 per group.

**Figure 4:**
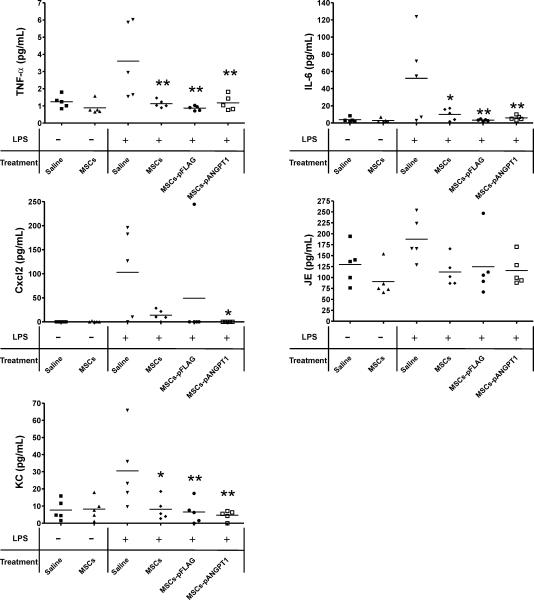
Levels of Proinflammatory Cytokines and Chemokines in Lung Homogenate Cytokine (TNF-α and IL6) and chemokine (Cxcl2, JE [murine homolog of human CCL2], and KC [murine IL8 homolog]) levels in lung homogenates were measured by multiplex immunoassay. Group comparisons were analyzed by one-way ANOVA with Dunnett post hoc test. *p < 0.05 and **p < 0.01, LPS/saline versus each treated group (MSCs, MSCs-pFLAG, or MSCs-pANGPT1). *n* = 5 per group.

To further evaluate the anti-inflammatory actions by MSCs and MSCs-pANGPT1, levels of proinflammatory cytokines and chemokines were measured in BAL fluid collected from animals. Proinflammatory cytokines (IFN-γ, TNF-α, IL6, and IL1β) were all elevated in BAL fluid in response to LPS challenge compared with naïve animals receiving saline (Figure 3). Treatment with MSCs alone variably decreased the levels of proinflammatory cytokines, while treatment with MSCs-pANGPT1 dramatically reduced cytokine levels to the baseline values observed in naïve mice. LPS instillation also increased the levels of Cxcl2, JE (the murine homolog of human CCL2), and KC (the murine homolog of human IL8) in BAL fluid, whereas treatment with MSCs, and to a greater extent MSCs-pANGPT1, attenuated these increases. Similarly, LPS-induced cytokine and chemokine levels in whole lung homogenates were significantly reduced by treatment with MSCs or MSCs-pANGPT1 (Figure 4), however, in this case MSCs alone did not differ significantly from MSCs-pANGPT1 in reducing proinflammatory cytokine and chemokine levels. Discrepancies in BAL cytokine levels between animals that received non- or null-transfected MSCs were occasionally noted, likely reflecting biological variability, since mice receiving null-transfected MSCs usually showed results similar to those in mice that received nontransfected MSCs. No detectable differences in cytokine and chemokine levels in plasma were observed among different treatment groups 3 d after treatment, suggesting that intratracheal LPS instillation induced localized inflammation in the lungs, and did not result in prolonged systemic inflammation in our model (unpublished data).

### Effect of MSCs and MSCs-pANGPT1 on LPS-Induced Lung Permeability

**Figure 5:**
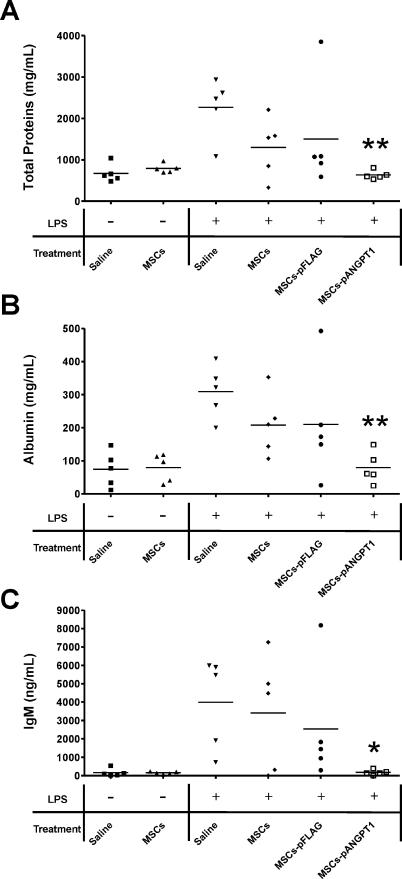
Effect of MSCs and MSCs-pANGPT1 on LPS-Induced ALI Therapeutic efficacy was assessed by measurement of total protein, albumin, and IgM (biomarkers of pulmonary vascular leakage resulting from disruption of the alveolar–capillary membrane barrier) in BAL fluid. (A) Total protein concentration was measured by Bradford assay (A); albumin was measured using a mouse-specific albumin ELISA (B); and IgM was measured using a mouse IgM ELISA kit (C). Group comparisons were analyzed by one-way ANOVA with Dunnett post hoc test. *p < 0.05 and **p < 0.01, LPS/saline versus each treated group (MSCs, MSCs-pFLAG, or MSCs-pANGPT1). *n* = 5 per group.

Concentrations of total protein, albumin, and IgM were assayed on collected BAL fluid to evaluate the integrity of the alveolar–capillary membrane barrier and assess pulmonary vascular leakage as a marker for ALI. These parameters of vascular leak were markedly increased (total protein, 3-fold; albumin, 4-fold; and IgM, 25-fold) in BAL fluid 3 d after LPS instillation compared to naïve mice. Whereas treatment with MSCs alone partially reduced total protein, albumin, and IgM levels (Figure 5), treatment with MSCs-pANGPT1 restored these lung injury indicators to levels not different from naïve control mice (*p* < 0.01 for total proteins and albumin, and *p* < 0.05 for IgM compared to LPS/saline group, respectively). To assess apoptosis in LPS-induced ALI mice with or without treatment, caspase 3 and 7 activities were measured in lung homogenate samples. Activities of caspase 3 and 7 in mouse lungs tended to be increased 3 d after animals received intratracheal instillation of LPS, which was normalized only in mice that received MSCs-pANGPT1 treatment (Figure S2; detailed method described in Protocol S1).

### Persistence of MSCs in Mice with or without LPS-Induced ALI

**Figure 6:**
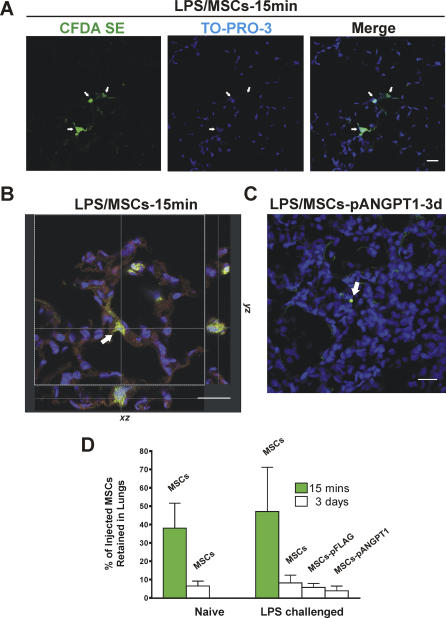
Retention of Injected MSCs in Mice With or Without LPS-Induced ALI MSCs were labeled with the cell tracing dye CFDA SE (green) prior to injection. Nuclei were stained with TO-PRO-3 (blue). Scale bars in photomicrographs = 20 μm. White arrows indicate labeled MSCs. (A) Labeled MSCs were observed in 5 μm, PFA-fixed lung sections from LPS-injured mice sacrificed at 15 min (initial retention). Image obtained with a Leica laser scanning confocal microscope with a 20× objective. (B) A three-dimensional lung section from an animal that received labeled MSCs. Lung was inflated, stored in OCT, and cut into 50 μm thick sections. Z-series images (30 sections, total thickness of the tissue scanned = 17.71 μm) were collected with a 63× oil objective and projected in different axes, as shown. Red autofluorescence shows outline of the alveolus. (C) pANGPT1-transfected MSCs labeled with the green fluorescent cell tracker CFDA SE were observed in lung section from LPS-injured mice killed at 3 d. Photomicrographs were taken in z-axis with a 20× objective, then images were stacked using Leica confocal software. (D) Lung lobes (left upper and all right lobes) from each animal were enzyme-digested into single cells before MSCs were counted by flow cytometry. *n* = 5 per group.

Retention of MSCs in the lung after central venous injection was verified by confocal microscopy and flow cytometry. MSCs labeled with the green fluorescent cell tracker CFDA SE [56] were observed in lung sections from both naïve and LPS-challenged mice sacrificed at 15 min (initial retention, Figure 6A). Although labeled cells could still be detected 3 d after injection, they were far less abundant (Figure 6C). No cell-specific green fluorescence was observed in sections from animals that did not receive CFDA SE-labeled cells (unpublished figure). To confirm that the green fluorescence observed was indeed from an intact cell, a z-series using laser scanning confocal microscopy was performed showing blue nuclear staining surrounded by green fluorescence by CFDA SE labeling (Figure 6B). The percentage of the injected MSCs retained in the lungs was quantified by flow cytometry following dispase lung digestion. An average 47% of injected cells were found in the lungs shortly after MSCs delivery in LPS-challenged mouse lungs comparing to 38% in naïve mice, though the difference was not statistically significant. Regardless of lung injury, the majority of MSCs were lost from the lung after 3 d, leaving less than 8% of cells remaining (Figure 6D).

## Discussion

In the present study, we demonstrate in a murine model of ALI that administration of MSCs to the pulmonary circulation partially prevented LPS-induced lung inflammation, whereas treatment with pANGPT1-transfected MSCs resulted in further improvement in both alveolar inflammation and permeability. These findings have potentially important implications for the treatment of ARDS, a clinical syndrome resulting from ALI, which represents an important therapeutic problem contributing to high morbidity and mortality of critically ill patients.

Cell-based gene transfer can overcome some of the limitations of direct gene therapy for pulmonary diseases [57], achieving selective and durable transgene expression in the lung [48–51,58]. Since stem and progenitor cells have also been reported to restore function of damaged tissue in various preclinical disease models [47,48], it follows that combining stem cell therapy with gene therapy might provide additive benefits. Indeed, we have reported that endothelial progenitor cells engineered to overexpress endothelial NO synthase were significantly more effective than endothelial progenitor cells alone in a monocrotaline model of vascular injury, producing near complete reversal of established pulmonary hypertension [48].

The choice of the most appropriate therapeutic gene is a critical determinant of the potential efficacy of a gene therapy strategy, and requires a detailed understanding of the pathogenic mechanisms underlying the target disorder. ARDS is a complex clinical syndrome that is initiated by injury to the lung, often in the setting of pneumonia or sepsis. Some of the early features of ARDS can be reproduced by administration of bacterial endotoxin (LPS), which acts via TLR4 (Toll-like receptor 4) to induce the expression of inflammatory cytokines and chemokines and up-regulate leukocyte adhesion molecules, resulting in EC activation [5–9,59]. Recently, it has been observed that activation of ECs in response to inflammatory cytokines is tightly regulated by ANGPT1. ANGPT1, the principal agonist of TEK, is an EC survival and vascular stabilization factor that maintains endothelial permeability in the normal postnatal vasculature [39–42]. In contrast, ANGPT2, which binds with equal affinity, has been described as a TEK antagonist, inhibiting receptor activation in response to ANGPT1 [60]. Fiedler et al. showed that ANGPT2 overexpression sensitized ECs to inflammatory stimuli and potentiated the up-regulation of adhesion molecules such as ICAM1 and VCAM1 in response to suboptimal doses of TNF-α [61]. Circulating levels of ANGPT2 were found to be elevated in the plasma of patients suffering from sepsis, and the administration of ANGPT2 led to disruption of endothelial barrier both in vitro and in a mouse model in vivo [62]. Moreover, ANGPT2 expression was recently observed to be increased in alveolar epithelial cells in response to hyperoxia-induced ALI, whereas ANGPT1 expression was reduced [63].

Therefore, a therapeutic strategy to restore the balance between ANGPT1 and ANGPT2 expression may be useful in modulating endothelial activation and inflammation in response to ALI. Systemic pretreatment with an adenoviral construct containing *ANGPT1* by Witzenbichler et al. was reported to improve hemodynamics, reduce adhesion molecule expression and prolong survival in mice with endotoxic shock [64]. Similarly, we have shown that fibroblasts transfected with *ANGPT1* were moderately effective in reducing ALI in rats when given one day prior to LPS, attenuating airspace inflammation by 60% and reducing expression of endothelial-selective adhesion molecules [65]. However, in these studies gene transfer was performed prior to lung injury, and it is uncertain whether post-treatment strategies, which are more relevant for clinical therapy, would have been equally effective. Furthermore, the Witzenbichler study used intraperitoneal administration of endotoxin and systemic gene therapy [64], and it is not possible to differentiate local actions of ANGPT1 on lung inflammation from its actions in the peripheral circulation.

The observed therapeutic effect of MSCs alone in ALI was not entirely unexpected, since previous reports have suggested that MSCs can inhibit the activation of dendritic cells, T lymphocytes, and natural killer cells, reducing the secretion of inflammatory cytokines (i.e., TNF-α, IFN-γ), increasing the release of anti-inflammatory cytokines (i.e., IL10, IL4), and inducing a tolerant phenotype [66,67]. The immunomodulatory effects of MSCs have been studied in a number of inflammatory states such as graft-versus-host disease [68] and autoimmune encephalomyelitis [69]. It has also been suggested that the immunosuppressive activity of MSCs, together with their lack of expression of class II major histocompatibility complex antigens, may confer an “immune-privileged” state in the context of allogenic transplantation, which could greatly simplify their therapeutic use. Indeed, in early clinical trials, the use of cultured allogenic MSCs was shown to be well tolerated, and did not induce a detectable immune response when transplanted into an unrelated recipient [30,70].

In the present study, there was an additional effect of ANGPT1 overexpressing MSCs compared with MSCs alone, not only on the extravasation of plasma proteins and inflammatory cells, but also on the levels of various inflammatory cytokines and chemokines in the BAL fluid. Interestingly, this additive effect was not apparent when these mediators were measured in whole-lung homogenates. ANGPT1 acts on TEK receptors, which are largely restricted to the endothelium. Thus, it is likely that ANGPT1 would have a preferential effect on the vascular endothelium, reducing inflammatory cell trafficking and plasma protein exudation into the alveolar space, and possibly inhibiting EC apoptosis as well. In contrast, effects of LPS on the airway epithelium or resident alveolar macrophages may not be directly modulated by ANGPT1. However, it should be noted that MSCs alone resulted in a near complete reduction of tissue cytokine expression, with the exception of Cxcl2, which in fact was further reduced in the group receiving with pANGPT1-transfected MSCs. It is also of interest that near complete normalization of protein and inflammatory cell exudation into the alveolus after intratracheal LPS instillation was achieved with MSCs-pANGPT1 delivered to the pulmonary circulation, despite the fact that alveolar permeability is determined by the functional integrity of both the alveolar epithelium and the alveolar endothelium, which together constitute the alveolar–capillary membrane barrier of the lung [1]. It has been well documented that LPS-induced ALI is associated with up-regulation of adhesion molecules on the endothelial surface [71]. Leukocyte adhesion to the activated endothelium is a critical determinant of the alveolar inflammatory response, leading to transmigration of inflammatory cells into the airspace and possible exacerbation of epithelial injury and increased pulmonary vascular leak [71]. Thus, the synergistic effect of *ANGPT1* gene transfer may be attributed to its ability to reduce EC activation and thus block the amplification of lung injury that is dependent on the recruitment of leukocytes from the pulmonary circulation into the injured lung.

On a separate note, we were unable to detect a significant increase in human ANGPT1 proteins in the lung tissue, BAL fluid, or plasma (unpublished data) after injection of pANGPT1-transfected MSCs. We believe that this is due to the lack of specific antibodies to human ANGPT1 that can reliably detect the human *ANGPT1* transgene product above the “noise” of the endogenous mouse proteins. In a separate study in which mice were allowed to survive up to 2 wk, a 20% mortality (up to 9 d) was observed in the LPS/saline group. No animals died in either the LPS/MSCs group or the LPS/MSCs-pANGPT1 group (unpublished data). We have also examined the effect of MSCs and MSCs-pANGPT1 at earlier time points (unpublished data). There was no difference in the total cell counts in BAL fluid in LPS-injured mice that received treatment with either MSCs or MSCs-pANGPT1, compared to the LPS/saline group (1 d after LPS injury). However, 2 d after LPS injury and treatment, there was a significant decrease in the total cell count in BAL fluid from mice that received MSCs and MSCs-pANGPT1 compared to controls, though the difference between those two groups was not significant. Preliminary data measuring the systemic level of IL1β suggested that plasma IL1β level peaked in LPS-injured mice (1 d after LPS) without treatment (LPS/saline mice), while most mice that had received treatment (LPS/MSCs or LPS/MSCs-pANGPT1) had lower or undetectable levels. Plasma IL1β rapidly declined in LPS-injured mice 2 d after injury. Mice that had received treatment (MSCs or MSCs-pANGPT1) had lower or undetectable levels.

We also examined whether the observed therapeutic effects depended on the retention of MSCs. A slightly higher proportion of cells were retained in injured lungs 15 minutes after LPS exposure; however, the difference between injured versus uninjured lung was not significant, and in both cases the majority of cells were lost from the lung by 3 d. Our results are in agreement with those of Beckett et al., who reported no significant improvement in lung engraftment of bone marrow-derived cells following endotoxin- or NO_2_-induced lung injury together with irradiation [72]. However, other studies have reported greater cell persistence after certain forms of injury [26,73–75]. Nonetheless, it is clear that the benefit seen in response to cell-based therapy in the LPS injury model did not require a high level of long-term persistence of transplanted MSCs. This observation is consistent with a number of reports of bone marrow cell therapy following myocardial infarction [76,77], which point to an important role for paracrine actions of transplanted cells on neovascularization and tissue healing. However, the precise mechanism by which genetically engineered MSCs confer a therapeutic benefit in the model of ALI remains to be determined.

### Study Limitations

It is important to recognize certain limitations of this study. First of all, the LPS-induced model of ALI cannot fully reproduce the complexity of clinical ALI/ARDS in human patients. Therefore, it will be necessary to reproduce these findings in more clinically relevant models, such as in animal models of sepsis and pneumonia. As well, cell therapy was given only 30 min after lung injury, and it will be important to define the therapeutic window of this intervention at later time points. Moreover, percentages of the retained cells in the lung after transplantation were fairly low, and future studies need to address the question of whether higher levels of MSCs incorporation would provide more therapeutic benefit in this and other models of ALI. A further limitation relates to the use syngeneic cells for cell therapy in the current study, which avoids the issue of host immune response. However, given the growing literature that these MSCs may be “immune-privileged,” it will be important in future work to explore whether the use of allogenic MSCs will have the same beneficial effects in this ALI model. The translation of these promising results into an effective new therapy for ARDS in patients will require, at the very least, that these limitations be addressed.

### Conclusion

In conclusion, we have shown that bone marrow-derived MSCs reduced the severity of ALI when administered into the pulmonary circulation of the LPS-injured mouse lung, possibly by virtue of their well-characterized immunomodulatory actions. However, this effect was greatly potentiated when these cells were engineered to overexpress the vasculoprotective factor ANGPT1, a specific inhibitor of inflammation and permeability in ECs. Our data show, to our knowledge for the first time, the synergistic action of combined cell and gene therapy for ALI, and may provide a basis for the development of an innovative approach for the prevention and treatment of ARDS, which continues to be a major cause of morbidity and mortality in critically ill patients.

## Supporting Information

Figure S1 Establishment of Optimal Conditions for Transfection of Murine MSCs MSCs were transfected by nuclear-targeting electroporation with pFLAG (null transfection control using high efficiency protocol, solid bar) or pANGPT1 (high cell survival or high efficiency protocol; grey or diagonal stripes bar, respectively). Supernatants were collected for 7 d and ANGPT1 proteins produced by nucleofected cells each day was quantified using ELISA. All plotted values represent net ANGPT1 proteins produced, in which the amount of ANGPT1 determined from a medium alone control was subtracted (239.74 pg/ml). All data are presented as mean ± SEM.
(219 KB TIF)

Figure S2 Caspase 3 and 7 Activity in Lung Homogenate Caspase 3 and Caspase 7 activities in mouse lung homogenates were measured by a commercially available kit. *n* = 5 per group.
(70 KB TIF)

Protocol S1 Detailed Information on Transfection of Murine MSCs and Measurement of Caspase-3/7 Activities in Lung Samples (20 KB DOC)

### Accession Numbers

The GenBank (http://www.ncbi.nlm.nih.gov/) accession numbers of the genes discussed in this paper are *ANGPT1* (GeneID 284); *ANGPT2* (GeneID 285); *TEK* (GeneID 7010).

## Abbreviations

ALI: acute lung injury
ANGPT1: human angiopoietin 1
ANGPT2: human angiopoietin 2
ARDS: acute respiratory distress syndrome
BAL: bronchoalveolar lavage
CFDA SE: carboxyfluorescein diacetate, succinimidyl ester
EC: endothelial cell
LPS: lipopolysaccharide
MSC: mesenchymal stem cell
MSCs-pANGPT1: MSCs transfected with plasmid containing human *ANGPT1* gene
pANGPT1: plasmid containing human *ANGPT1* gene
SEM: standard error of the mean
TEK: TEK tyrosine kinase

## References

[pmed-0040269-b001] Ware LB, Matthay MA (2000). The acute respiratory distress syndrome. N Engl J Med.

[pmed-0040269-b002] Goss CH, Brower RG, Hudson LD, Rubenfeld GD (2003). Incidence of acute lung injury in the United States. Crit Care Med.

[pmed-0040269-b003] Mendez JL, Hubmayr RD (2005). New insights into the pathology of acute respiratory failure. Curr Opin Crit Care.

[pmed-0040269-b004] Rubenfeld GD, Caldwell E, Peabody E, Weaver J, Martin DP (2005). Incidence and outcomes of acute lung injury. N Engl J Med.

[pmed-0040269-b005] Kitamura Y, Hashimoto S, Mizuta N, Kobayashi A, Kooguchi K (2001). Fas/FasL-dependent apoptosis of alveolar cells after lipopolysaccharide-induced lung injury in mice. Am J Respir Crit Care Med.

[pmed-0040269-b006] Matute-Bello G, Winn RK, Martin TR, Liles WC (2004). Sustained lipopolysaccharide-induced lung inflammation in mice is attenuated by functional deficiency of the Fas/Fas ligand system. Clin Diagn Lab Immunol.

[pmed-0040269-b007] Rojas M, Woods CR, Mora AL, Xu J, Brigham KL (2005). Endotoxin-induced lung injury in mice: Structural, functional, and biochemical responses. Am J Physiol Lung Cell Mol Physiol.

[pmed-0040269-b008] Altemeier WA, Matute-Bello G, Gharib SA, Glenny RW, Martin TR (2005). Modulation of lipopolysaccharide-induced gene transcription and promotion of lung injury by mechanical ventilation. J Immunol.

[pmed-0040269-b009] Gharib SA, Liles WC, Matute-Bello G, Glenny RW, Martin TR (2006). Computational identification of key biological modules and transcription factors in acute lung injury. Am J Respir Crit Care Med.

[pmed-0040269-b010] Ware LB, Matthay MA (2001). Alveolar fluid clearance is impaired in the majority of patients with acute lung injury and the acute respiratory distress syndrome. Am J Respir Crit Care Med.

[pmed-0040269-b011] Guidot DM, Folkesson HG, Jain L, Sznajder JI, Pittet JF (2006). Integrating acute lung injury and regulation of alveolar fluid clearance. Am J Physiol Lung Cell Mol Physiol.

[pmed-0040269-b012] Crimi E, Slutsky AS (2004). Inflammation and the acute respiratory distress syndrome. Best Pract Res Clin Anaesthesiol.

[pmed-0040269-b013] The Acute Respiratory Distress Syndrome Network (2000). Ventilation with lower tidal volumes as compared with traditional tidal volumes for acute lung injury and the acute respiratory distress syndrome. N Engl J Med.

[pmed-0040269-b014] Matthay MA, Zimmerman GA, Esmon C, Bhattacharya J, Coller B (2003). Future research directions in acute lung injury: Summary of a National Heart, Lung, and Blood Institute working group. Am J Respir Crit Care Med.

[pmed-0040269-b015] Mehta D, Bhattacharya J, Matthay MA, Malik AB (2004). Integrated control of lung fluid balance. Am J Physiol Lung Cell Mol Physiol.

[pmed-0040269-b016] Slutsky AS, Hudson LD (2006). PEEP or no PEEP—Lung recruitment may be the solution. N Engl J Med.

[pmed-0040269-b017] Koc ON, Lazarus HM (2001). Mesenchymal stem cells: Heading into the clinic. Bone Marrow Transplant.

[pmed-0040269-b018] Barry FP, Murphy JM (2004). Mesenchymal stem cells: Clinical applications and biological characterization. Int J Biochem Cell Biol.

[pmed-0040269-b019] Horwitz EM (2003). Stem cell plasticity: The growing potential of cellular therapy. Arch Med Res.

[pmed-0040269-b020] Dominici M, Hofmann TJ, Horwitz EM (2001). Bone marrow mesenchymal cells: Biological properties and clinical applications. J Biol Regul Homeost Agents.

[pmed-0040269-b021] Baksh D, Song L, Tuan RS (2004). Adult mesenchymal stem cells: Characterization, differentiation, and application in cell and gene therapy. J Cell Mol Med.

[pmed-0040269-b022] Stripp BR, Shapiro SD (2006). Stem cells in lung disease, repair, and the potential for therapeutic interventions: State-of-the-art and future challenges. Am J Respir Cell Mol Biol.

[pmed-0040269-b023] Prockop DJ (1997). Marrow stromal cells as stem cells for nonhematopoietic tissues. Science.

[pmed-0040269-b024] Dominici M, Le Blanc K, Mueller I, Slaper-Cortenbach I, Marini F (2006). Minimal criteria for defining multipotent mesenchymal stromal cells. The International Society for Cellular Therapy position statement. Cytotherapy.

[pmed-0040269-b025] Ortiz LA, Gambelli F, McBride C, Gaupp D, Baddoo M (2003). Mesenchymal stem cell engraftment in lung is enhanced in response to bleomycin exposure and ameliorates its fibrotic effects. Proc Natl Acad Sci U S A.

[pmed-0040269-b026] Yamada M, Kubo H, Kobayashi S, Ishizawa K, Numasaki M (2004). Bone marrow-derived progenitor cells are important for lung repair after lipopolysaccharide-induced lung injury. J Immunol.

[pmed-0040269-b027] Kotton DN, Ma BY, Cardoso WV, Sanderson EA, Summer RS (2001). Bone marrow-derived cells as progenitors of lung alveolar epithelium. Development.

[pmed-0040269-b028] Grove JE, Lutzko C, Priller J, Henegariu O, Theise ND (2002). Marrow-derived cells as vehicles for delivery of gene therapy to pulmonary epithelium. Am J Respir Cell Mol Biol.

[pmed-0040269-b029] Koc ON, Day J, Nieder M, Gerson SL, Lazarus HM (2002). Allogeneic mesenchymal stem cell infusion for treatment of metachromatic leukodystrophy (MLD) and Hurler syndrome (MPS-IH). Bone Marrow Transplant.

[pmed-0040269-b030] Horwitz EM, Gordon PL, Koo WK, Marx JC, Neel MD (2002). Isolated allogeneic bone marrow-derived mesenchymal cells engraft and stimulate growth in children with osteogenesis imperfecta: Implications for cell therapy of bone. Proc Natl Acad Sci U S A.

[pmed-0040269-b031] Krampera M, Cosmi L, Angeli R, Pasini A, Liotta F (2006). Role for interferon-gamma in the immunomodulatory activity of human bone marrow mesenchymal stem cells. Stem Cells.

[pmed-0040269-b032] Frank MH, Sayegh MH (2004). Immunomodulatory functions of mesenchymal stem cells. Lancet.

[pmed-0040269-b033] Le Blanc K (2003). Immunomodulatory effects of fetal and adult mesenchymal stem cells. Cytotherapy.

[pmed-0040269-b034] Di Nicola M, Carlo-Stella C, Magni M, Milanesi M, Longoni PD (2002). Human bone marrow stromal cells suppress T-lymphocyte proliferation induced by cellular or nonspecific mitogenic stimuli. Blood.

[pmed-0040269-b035] Neuringer IP, Randell SH (2004). Stem cells and repair of lung injuries. Respir Res.

[pmed-0040269-b036] Davis S, Aldrich TH, Jones PF, Acheson A, Compton DL (1996). Isolation of angiopoietin-1, a ligand for the TIE2 receptor, by secretion-trap expression cloning. Cell.

[pmed-0040269-b037] Suri C, Jones PF, Patan S, Bartunkova S, Maisonpierre PC (1996). Requisite role of angiopoietin-1, a ligand for the TIE2 receptor, during embryonic angiogenesis. Cell.

[pmed-0040269-b038] Thurston G, Rudge JS, Ioffe E, Papadopoulos N, Daly C (2005). The anti-inflammatory actions of angiopoietin-1. EXS.

[pmed-0040269-b039] Gamble JR, Drew J, Trezise L, Underwood A, Parsons M (2000). Angiopoietin-1 is an antipermeability and anti-inflammatory agent in vitro and targets cell junctions. Circ Res.

[pmed-0040269-b040] Thurston G, Rudge JS, Ioffe E, Zhou H, Ross L (2000). Angiopoietin-1 protects the adult vasculature against plasma leakage. Nat Med.

[pmed-0040269-b041] Baffert F, Le T, Thurston G, McDonald DM (2006). Angiopoietin-1 decreases plasma leakage by reducing number and size of endothelial gaps in venules. Am J Physiol Heart Circ Physiol.

[pmed-0040269-b042] Thurston G, Suri C, Smith K, McClain J, Sato TN (1999). Leakage-resistant blood vessels in mice transgenically overexpressing angiopoietin-1. Science.

[pmed-0040269-b043] Kwak HJ, So JN, Lee SJ, Kim I, Koh GY (1999). Angiopoietin-1 is an apoptosis survival factor for endothelial cells. FEBS Lett.

[pmed-0040269-b044] Papapetropoulos A, Garcia-Cardena G, Dengler TJ, Maisonpierre PC, Yancopoulos GD (1999). Direct actions of angiopoietin-1 on human endothelium: Evidence for network stabilization, cell survival, and interaction with other angiogenic growth factors. Lab Invest.

[pmed-0040269-b045] Chen JX, Chen Y, DeBusk L, Lin W, Lin PC (2004). Dual functional roles of Tie-2/angiopoietin in TNF-alpha-mediated angiogenesis. Am J Physiol Heart Circ Physiol.

[pmed-0040269-b046] Papapetropoulos A, Fulton D, Mahboubi K, Kalb RG, O'Connor DS (2000). Angiopoietin-1 inhibits endothelial cell apoptosis via the Akt/survivin pathway. J Biol Chem.

[pmed-0040269-b047] Kanki-Horimoto S, Horimoto H, Mieno S, Kishida K, Watanabe F (2006). Implantation of mesenchymal stem cells overexpressing endothelial nitric oxide synthase improves right ventricular impairments caused by pulmonary hypertension. Circulation.

[pmed-0040269-b048] Zhao YD, Courtman DW, Deng Y, Kugathasan L, Zhang Q (2005). Rescue of monocrotaline-induced pulmonary arterial hypertension using bone marrow-derived endothelial-like progenitor cells: Efficacy of combined cell and eNOS gene therapy in established disease. Circ Res.

[pmed-0040269-b049] Campbell AI, Zhao Y, Sandhu R, Stewart DJ (2001). Cell-based gene transfer of vascular endothelial growth factor attenuates monocrotaline-induced pulmonary hypertension. Circulation.

[pmed-0040269-b050] Kugathasan L, Dutly AE, Zhao YD, Deng Y, Robb MJ (2005). Role of angiopoietin-1 in experimental and human pulmonary arterial hypertension. Chest.

[pmed-0040269-b051] Zhao YD, Campbell AI, Robb M, Ng D, Stewart DJ (2003). Protective role of angiopoietin-1 in experimental pulmonary hypertension. Circ Res.

[pmed-0040269-b052] Peister A, Mellad JA, Larson BL, Hall BM, Gibson LF (2004). Adult stem cells from bone marrow (MSCs) isolated from different strains of inbred mice vary in surface epitopes, rates of proliferation, and differentiation potential. Blood.

[pmed-0040269-b053] Harris RG, Herzog EL, Bruscia EM, Grove JE, Van Arnam JS (2004). Lack of a fusion requirement for development of bone marrow-derived epithelia. Science.

[pmed-0040269-b054] Matute-Bello G, Winn RK, Jonas M, Chi EY, Martin TR (2001). Fas (CD95) induces alveolar epithelial cell apoptosis in vivo: Implications for acute pulmonary inflammation. Am J Pathol.

[pmed-0040269-b055] Dhanireddy S, Altemeier WA, Matute-Bello G, O'Mahony DS, Glenny RW (2006). Mechanical ventilation induces inflammation, lung injury, and extra-pulmonary organ dysfunction in experimental pneumonia. Lab Invest.

[pmed-0040269-b056] Dooner M, Cerny J, Colvin G, Demers D, Pimentel J (2004). Homing and conversion of murine hematopoietic stem cells to lung. Blood Cells Mol Dis.

[pmed-0040269-b057] Weiss DJ (2002). Delivery of gene transfer vectors to lung: Obstacles and the role of adjunct techniques for airway administration. Mol Ther.

[pmed-0040269-b058] Campbell AI, Kuliszewski MA, Stewart DJ (1999). Cell-based gene transfer to the pulmonary vasculature: Endothelial nitric oxide synthase overexpression inhibits monocrotaline-induced pulmonary hypertension. Am J Respir Cell Mol Biol.

[pmed-0040269-b059] Fan J, Frey RS, Malik AB (2003). TLR4 signaling induces TLR2 expression in endothelial cells via neutrophil NADPH oxidase. J Clin Invest.

[pmed-0040269-b060] Maisonpierre PC, Suri C, Jones PF, Bartunkova S, Wiegand SJ (1997). Angiopoietin-2, a natural antagonist for Tie2 that disrupts in vivo angiogenesis. Science.

[pmed-0040269-b061] Fiedler U, Reiss Y, Scharpfenecker M, Grunow V, Koidl S (2006). Angiopoietin-2 sensitizes endothelial cells to TNF-alpha and has a crucial role in the induction of inflammation. Nat Med.

[pmed-0040269-b062] Parikh SM, Mammoto T, Schultz A, Yuan HT, Christiani D (2006). Excess circulating angiopoietin-2 may contribute to pulmonary vascular leak in sepsis in humans. PLoS Med.

[pmed-0040269-b063] Bhandari V, Choo-Wing R, Lee CG, Zhu Z, Nedrelow JH (2006). Hyperoxia causes angiopoietin 2-mediated acute lung injury and necrotic cell death. Nat Med.

[pmed-0040269-b064] Witzenbichler B, Westermann D, Knueppel S, Schultheiss HP, Tschope C (2005). Protective role of angiopoietin-1 in endotoxic shock. Circulation.

[pmed-0040269-b065] McCarter SD, Mei SH, Lai PF, Zhang Q, Parker CH (2007). Cell-based angiopoietin-1 gene therapy for acute lung injury. Am J Respir Crit Care Med.

[pmed-0040269-b066] Aggarwal S, Pittenger MF (2005). Human mesenchymal stem cells modulate allogeneic immune cell responses. Blood.

[pmed-0040269-b067] Batten P, Sarathchandra P, Antoniw JW, Tay SS, Lowdell MW (2006). Human mesenchymal stem cells induce T cell anergy and downregulate T cell allo-responses via the TH2 pathway: Relevance to tissue engineering human heart valves. Tissue Eng.

[pmed-0040269-b068] Le Blanc K, Rasmusson I, Sundberg B, Gotherstrom C, Hassan M (2004). Treatment of severe acute graft-versus-host disease with third party haploidentical mesenchymal stem cells. Lancet.

[pmed-0040269-b069] Zappia E, Casazza S, Pedemonte E, Benvenuto F, Bonanni I (2005). Mesenchymal stem cells ameliorate experimental autoimmune encephalomyelitis inducing T-cell anergy. Blood.

[pmed-0040269-b070] Ryan JM, Barry FP, Murphy JM, Mahon BP (2005). Mesenchymal stem cells avoid allogeneic rejection. J Inflamm (Lond).

[pmed-0040269-b071] Zimmerman GA, Albertine KH, Carveth HJ, Gill EA, Grissom CK (1999). Endothelial activation in ARDS. Chest.

[pmed-0040269-b072] Beckett T, Loi R, Prenovitz R, Poynter M, Goncz KK (2005). Acute lung injury with endotoxin or NO_2_ does not enhance development of airway epithelium from bone marrow. Mol Ther.

[pmed-0040269-b073] Anjos-Afonso F, Siapati EK, Bonnet D (2004). In vivo contribution of murine mesenchymal stem cells into multiple cell-types under minimal damage conditions. J Cell Sci.

[pmed-0040269-b074] Theise ND, Henegariu O, Grove J, Jagirdar J, Kao PN (2002). Radiation pneumonitis in mice: A severe injury model for pneumocyte engraftment from bone marrow. Exp Hematol.

[pmed-0040269-b075] Herzog EL, Van Arnam J, Hu B, Krause DS (2006). Threshold of lung injury required for the appearance of marrow-derived lung epithelia. Stem Cells.

[pmed-0040269-b076] van Laake L, Hassink R, Doevendans P, Mummery C (2006). Heart repair and stem cells. J Physiol.

[pmed-0040269-b077] Fukuda K, Yuasa S (2006). Stem cells as a source of regenerative cardiomyocytes. Circ Res.

