# Supplementary material for: Prevention of LPS-Induced Acute Lung Injury in Mice by Mesenchymal Stem Cells Overexpressing Angiopoietin 1
Source: PLoS Med. 2007 Sep 4;4(9):e269. doi: 10.1371/journal.pmed.0040269 (PMC1961632; doi:10.1371/journal.pmed.0040269)
Supplement: Supplementary file 1 [file pmed.0040269.sd001.doc]

**SUPPLEMENTARY METHODS**

Nuclear-targeting electroporation (Amaxa, Gaithersburg, MD) was used to transfect MSCs with Ang1 plasmid or empty vector plasmid, following manufacture’s protocol. Human Ang1 protein expression was verified by ELISA (R&D Systems, Minneapolis, MN). For the *in vivo* experiment, the high efficiency protocol was employed to transfect MSCs before injection. Caspase-3/7 activities were measured from lung homogenate samples with commercially available kit (Promega, Madison, WI) according to manufacture’s instructions. All results were normalized (25 µg total protein per sample) with individual protein concentration measured from lung homogenate sample by Bradford assay (Bio-Rad, Hercules, CA).
